# Cultivable endophytic fungal community associated with the karst endemic plant *Nervilia fordii* and their antimicrobial activity

**DOI:** 10.3389/fmicb.2022.1063897

**Published:** 2022-11-24

**Authors:** Ya-Qin Zhou, Shao-Chang Yao, Jie Wang, Xin-Yi Xie, Xiao-Ming Tan, Rong-Shao Huang, Xin-Feng Yang, Yong Tan, Li-Ying Yu, Peng Fu

**Affiliations:** ^1^College of Pharmacy, Guangxi University of Chinese Medicine, Nanning, China; ^2^Guangxi Key Laboratory of Medicinal Resources Conservation and Genetic Improvement, Guangxi Botanical Garden of Medicinal Plants, Nanning, China; ^3^Guangxi Zhuang Yao Key Laboratory of Medicine, Guangxi University of Chinese Medicine, Nanning, China

**Keywords:** endophytic fungi, antimicrobial activity, culturable fungal diversity, *N. fordii*, tissue specificity, *P. macrosclerotiorum*, methyl chloroacetate

## Abstract

Endophytic fungi from medicinal plants with specific pharmacological functions attract much attention to provide the possibility of discovering valuable natural drugs with novel structures and biological activities. *Nervilia fordii* is a rare and endangered karst endemic plant that is used as medicine and food homology in Guangxi, China. These plants have been reported to have antimicrobial, antitumor, antiviral, and anti-inflammatory activities. However, few studies have focused on the diversity and antibacterial activity of endophytic fungi from *N. fordii*. In the present study, 184 endophytic fungi were isolated from the healthy tissues of *N. fordii*, and their molecular diversity and antimicrobial activities were analyzed for the first time. These fungi were categorized into 85 different morphotypes based on the morphological characteristics and the similarity between the target sequence and the reference sequence in the GenBank database. With the exception of 18 unidentified fungi, the fungal isolates belonged to at least 2 phyla, 4 classes, 15 orders, 45 known genera, and 45 different species, which showed high abundance, rich diversity, and obvious tissue specificity. All isolates were employed to screen for their antimicrobial activities *via* the agar diffusion method against *Escherichia coli*, *Staphylococcus aureus*, and *Candida tropicalis*. Among these endophytes, eight strains (9.41%) displayed inhibitory activity against *E. coli*, 11 strains (12.94%) against *S. aureus*, and two strains (2.35%) against *C. tropicalis*, to some extent. In particular, our study showed for the first time that the fungal agar plugs of *Penicillium macrosclerotiorum* 1151# exhibited promising antibacterial activity against *E. coli* and *S. aureus*. Moreover, the ethyl acetate (EA) extract of *P. macrosclerotiorum* 1151# had antibacterial effects against *E. coli* and *S. aureus* with a minimum inhibitory concentration (MIC) of 0.5 mg ml^–1^. Further research also confirmed that one of the antimicrobial compounds of *P. macrosclerotiorum* 1151# was methyl chloroacetate and exhibited excellent antibacterial activity against *E. coli* and *S. aureus* up to 1.71-fold and 1.13-fold compared with tetracycline (TET) (5 mg ml^–1^), respectively. Taken together, the present data suggest that various endophytic fungi of *N. fordii* could be exploited as sources of novel natural antimicrobial agents.

## Introduction

The emergence of novel coronavirus pathogens, monkeypox, and mucormycosis (Feldman and Anderson, 2021; Salem et al., 2022a), as well as the re-emergence of microbial diseases such as tuberculosis, whooping cough, and urinary tract infections, have posed an unprecedented threat to human lives and health (Rajivgandhi et al., 2016; Mahendra et al., 2022). In fact, antimicrobial resistance is the most serious obstacle to dealing with such threats. Unfortunately, no new antibiotics have been discovered in the last two decades, although the search for new antibiotics has never stopped (Rajivgandhi et al., 2020; Böttcher et al., 2021). Therefore, it is necessary to identify new antibacterial drugs to deal with emerging microbial diseases (Andryukov et al., 2019; Seukep et al., 2020).

Endophytic fungi are microorganisms that live within the cells or tissues of their host plants at a certain stage without causing obvious disease to plant tissues (Petrini et al., 1992). Studies have shown that endophytic fungi are prevalent in various ecosystems worldwide, with the exception of Antarctica (Guo, 2018). The number of endophytic fungi is conservatively estimated to be more than 1 million. However, about 95% of endophytic fungi have not been described, which means that many novel natural active ingredients may be discovered from endophytic fungi. In fact, some active substances, such as penicillin, cephalosporin, and ß-lactam antibiotics, produced by endophytic fungi have shown great economic value and application prospects in the development of antimicrobial drugs (Terfehr et al., 2017). In addition, biologists have also used fungi biological techniques to rapidly synthesize nanocrystals (Ag, Zn, Se, etc.) with antibacterial, antifungal, and anticancer activities (Hashem et al., 2022; Salem et al., 2022b; Shehabeldine et al., 2022a). Therefore, endophytic fungi provide the possibility of discovering valuable natural drugs with novel structures and biological activities (Elghaffar et al., 2022).

In recent years, many studies have found that endophytic fungi and their host plants have similar metabolite-synthesis pathways, which can produce the secondary metabolites of host plants. For example, *Taxomyces andreanae* isolated from *Taxus brevifolia* produces the anticancer component paclitaxel (Stierle et al., 1993), whereas *Phialocephala fortinii* isolated from *Rhodiola angusta* produces the antioxidant components salidroside and p-tyrosol (Cui et al., 2016). Therefore, endophytic fungi from medicinal plants with specific pharmacological functions have become a research hotspot.

**FIGURE 1:**
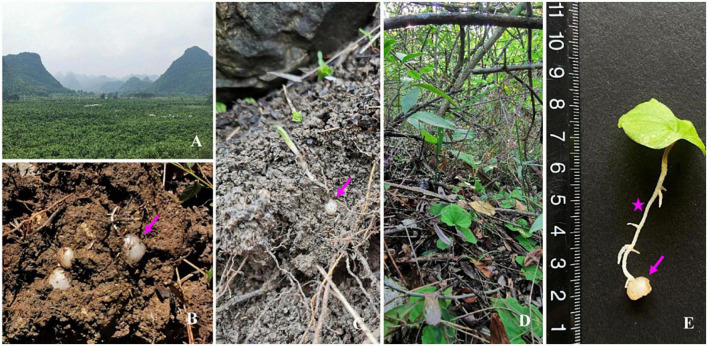
Habitat of *Nervilia fordii*. **(A)** *Nervilia fordii* often located in the limestone mountains of the karst area in Guangxi Province, China. **(B)** The corm is the living state of *N. fordii* in every cold season. **(C)** The corm (↑) of *N. fordii* always sprout and break through the soil when the temperature rises in every March or April. **(D)** A community of *N. fordii* were found under bushes of the karst. **(E)** The whole plant of *N. fordii* is composed of the leaves, roots (*), and corms (↑).

*Nervilia fordii* (Hance) Schltr. is a small, terrestrial, and short-lived perennial plant (Orchidaceae) that is endemic to the karst limestone mountains (Figures 1A–D) in Guangxi Province, China. The whole plant or the aerial part of *N. fordii* (Figure 1E) are used as premium dishes, herbal tea, and folk medicine (Qiu et al., 2013) because of their excellent pharmacological capacities, such as antimicrobial (Li et al., 2017; Xu et al., 2017), anticancer (Yao et al., 2021), antiviral (Tian et al., 2009), and anti-inflammatory (Yin et al., 2021) effects. In particular, this plant was widely used as a main ingredient in traditional Chinese medicine formulations to treat patients with severe acute respiratory syndrome in 2013 (Qiu et al., 2013). Further studies have reported that flavonoids, terpenes, sterols, volatile oils, and amino acids are the bioactive ingredients of *N. fordii* (Wei et al., 2016). However, to date, few studies have reported the diversity and antimicrobial activities of endophytic fungi associated with *N. fordii* plants. The aims of this present study were: (1) to provide the first evidence of the diversity, phylogeny, and taxonomic composition of culturable endophytic fungi isolated from *N. fordii* in the karst region; (2) to evaluate the potential antimicrobial activities of these endophytic fungi against Gram-negative bacteria, Gram-positive bacteria, and unicellular fungi using the agar diffusion method; and (3) to investigate the antimicrobial compounds of selected endophytic fungi exhibiting excellent antimicrobial capacities.

## Materials and methods

### Pant material collection

*Nervilia fordii* samples were collected from Yongfu county (altitude, 270 m; E109°66′, N24°94′) (Figure 1A) in Guangxi province, China. They were identified by Li-Ying Yu and voucher specimens were preserved in the herbarium of the Guangxi Botanical Garden of Medicinal Plants (voucher ID: SHNF20200618).

### Fungal isolation and cultivation

The procedures used for the surface sterilization of the *N. fordii* samples were according to the methods described by Tan et al. (2018). Briefly, the roots, corms, and leaves were separated from the plants and thoroughly washed under running tap water. Subsequently, surface sterilization was performed sequentially by soaking the plant materials in 70% ethanol (*v*/*v*) for 30 s, followed by soaking in 2.5% sodium hypochlorite (*v*/*v*) for 4–5 min; the materials were then rinsed with sterile distilled water three times. All sterilized materials were finally dried with sterile filter paper and then divided into segments using a sterilized scalpel. The segments of tissue were placed in Petri dishes containing Potato Dextrose Agar (PDA) medium with 50 μg ml^–1^ oxytetracycline and 50 μg ml^–1^ streptomycin (SM). The Petri dishes were then sealed with parafilm and incubated at 25 ± 2°C in the dark for up to 4 weeks to allow the isolation of slow growing endophytic fungi (Pecundo et al., 2021). The colonies that emerged from segments of tissue were periodically examined, and transferred in a timely manner to fresh antibiotic-free PDA medium by using the hyphal method to obtain the purified colonies of endophytic fungi. Then, all purified isolates were categorized and maintained at the Scientific Laboratory Center of the Guangxi University of Chinese Medicine.

### Molecular analysis of endophytic fungi

Fresh mycelia were used to extract DNA according to the instructions provided by E.Z.N.A.TM Fungal DNA Mini Kits (Omega Bio-tek, Norcross, GA, USA). The rDNA region, including the internal transcribed spacer 1, internal transcribed spacer 2, and 5.8s gene, was amplified by PCR using the following primer pair: ITS1 (5′–TCCGTAGGTGAACCTGCGG–3′) and ITS4 (5′–TCCTCCGCTTATTGATATGC–3′) (White et al., 1990). The PCR mixture (50 μl) included 25 μl of 2 × SanTaq PCR Mix (Sangon Biotech, Shanghai), 2 μl of each primer (5 μM), 10 μl of genomic DNA (50 ng⋅μl^–1^), and 11 μl of autoclaved double-distilled water. PCR amplification was performed in a thermal cycler (BioRad) as follows: initial denaturation at 94°C for 3 min; followed by 35 cycles of 94°C for 30 s, 55°C for 25 s, and 72°C for 30 s; and a final extension at 72°C for 7 min. All PCR products were visualized by electrophoresis on a 1.5% (wt/v) agarose gel in 1 × TBE buffer (40 mmol L^–1^ Tris; 1 mmol L^–1^ EDTA, pH 8.0). Then, the certified products were sent to Shanghai Shengon Company Ltd. (Shanghai, China) for sequencing. The sequence data from 85 representative culturable endophytic fungi obtained in this study were submitted to GenBank. The phylogenetic analysis was performed by the Neighbor-joining (NJ) method using Molecular evolutionary genetics analysis (MEGA) software (Tejesvi et al., 2011; Rajivgandhi et al., 2018a).

### Sequence accessions

The sequence data from the 85 fungal isolates were deposited in GenBank (the accession numbers are provided in Supplementary Table 1).

### Antimicrobial screening of fungal agar plug

The agar well diffusion method described by Gauchan et al. (2020) was used to evaluate the antimicrobial activity of the 85 fungal isolates against *Staphylococcus aureus* (ATCC 25923), *Escherichia coli* (ATCC 35401), and *Candida tropicalis* (ATCC 66029). First, the pre-cultured pathogenic indicator microorganism at approximately 10^7^ CFU ml^–1^ was inoculated into 20 ml of molten nutrient agar. The solidified culture medium was used to punch wells with a sterile cork borer (φ = 6 mm). A sterile inoculating needle was employed to remove the agar plugs. Subsequently, the fungal endophyte agar plugs (φ = 6 mm) with different concentrations of the fungal extract were placed into separate wells, respectively. The PDA agar plugs was used as the negative control. The plates were incubated at 37°C (*S. aureus* and *E. coli*) or 28°C (*C. tropicalis*) for 24 h and the diameter of the inhibition zone was measured using a vernier caliper. The experiments were performed in triplicate. Streptomycin and tetracycline (Sigma, USA) were employed as the positive controls.

Bacterial species *S. aureus* and *E. coli*, were pre-incubated at 37°C on LB medium [yeast extract, 0.5% (*w*/*v*); tryptone, 1% (*w*/*v*); NaCl, 0.5% (*w*/*v*); agar, 2%(*w*/*v*); and pH 7.2] periodically. The fungal pathogenic *C. tropicalis* was pre-incubated at 28°C on Sabouraud medium [peptone, 1% (*w*/*v*); glucose, 4% (*w*/*v*); agar, 2% (*w*/*v*); and pH 5.8] periodically. All fungal isolates were maintained on PDA (Difco) at 25°C for 2 weeks before the antimicrobial assay.

### Determination of antimicrobial composition from *Penicillium macrosclerotiorum* 1151#

The antimicrobial composition of the fungal isolate *Penicillium macrosclerotiorum* 1151#, which exhibited the highest antimicrobial activity, was determined by ^1^H Nuclear Magnetic Resonance (NMR) and ^13^C NMR. The specific experimental methods used were as follows.

#### Fermentation and extraction

*Penicillium macrosclerotiorum* 1151# was pre-cultured on PDA at 25 ± 2°C in the dark for 7 days. Four mycelial agar plugs (φ = 6 mm) collected from the edge of the fungal colony were inoculated into a 500-ml Erlenmeyer flask containing 120 g of sterile rice medium. The flasks were incubated at 25 ± 2°C in the dark for 2 weeks. The fungal fermentation product in the rice medium was extracted three times with 10-fold (w/v) petroleum ether (PET), ethyl acetate (EA), or *n*-butanol (*n*-BuOH) by ultrasonication for 1 h, to yield crude extraction solutions, respectively. The three crude extraction solutions were combined and filtered using Whatman No. 1 filter paper. The filtrate was further concentrated under reduced pressure (8 × 10^3^ Pa) to remove the organic solvent, and the concentrates were then volatilized in a water bath at 60°C, to obtain the dried crude extract.

#### *In vitro* antibacterial activity and minimum inhibitory concentration determination of ethyl acetate crude extract

The antibacterial activity of EA crude extract of *P. macrosclerotiorum* 1151# was evaluated *in vitro* against Gram-negative bacteria (*E. coli*) and Gram-positive bacteria (*S. aureu*s) using the agar well diffusion method as described by Shehabeldine et al. (2022b). In this study, each pre-cultured pathogenic bacteria at approximately 10^7^ CFU ml^–1^ was inoculated into 20 ml of sterilized Luria-Bertani (LB) plate. Wells of 6-mm diameter was made on the LB plates using a sterile cork borer. The well was loaded with 20 μL of EA crude extracts diluted in 10% (*v*/*v*) dimethyl sulfoxide (DMSO) at various concentrations (0.1, 0.5, 1.0, 2.5, 5, and 10 mg⋅ml^–1^). DMSO was used as negative control, and TET dissolved in 10% (*v*/*v*) DMSO was employed as positive controls. The plates were incubated at 37°C for 24 h, and the radius of the inhibition zone was measured with a vernier caliper. Minimal inhibitory concentration (MIC) was recorded as the lowest concentration of extract that inhibited the growth of *E. coli* or *S. aureus*. All experiments were performed in triplicate and repeated twice.

#### Separated and purification of most active compound

In order to analyze the most active compound, the EA crude extract was chromatographed on a D_101_ macroporous resin column using a gradient of PET:acetone. The most active fractions were assessed and further purified by semi-preparative HPLC (MeOH:H_2_O, 1:1, v/v), according to the antibacterial assays.

#### Structure determination of the compound by ^1^H and ^13^C-NMR

To confirm the structure of the separated compound, NMR spectra were recorded on a Bruker AM-400 spectrometer (Bruker Corporation, Fallanden, Switzerland), which was operated at 400 MHz for ^1^H NMR and 100 MHz for ^13^C NMR. The chemical shifts are given in δ (ppm) using TMS as the internal standard. HR-ESI-MS was measured on a Q-TOF Ultima GLOBAL GAA076 LC mass spectrometer. Semi-preparative HPLC was performed on a Shimadzu SPD-20A Liquid Chromatograph with an SPD-20A detector using a C_18_ column (5 μm, ϕ 250 mm × 20 mm, YMC-pack ODS-A, Agilent, 2 ml min^–1^).

### Statistical analyses

The Shannon–Wiener and Simpson indices were employed to compare the diversity of endophytic fungal communities among the leaves, roots, and corms of *N. fordii*. The two diversity indices were evaluated using the methods reported by Koukol et al. (2012). All antimicrobial experiments were performed in triplicate and the data are presented as the mean ± standard deviation. Statistical analyses were performed using SPSS 19.0 (SPSS Inc., Chicago, IL, USA).

## Results

### Phylogenetic analyses of culturable endophytic fungi

**FIGURE 2:**
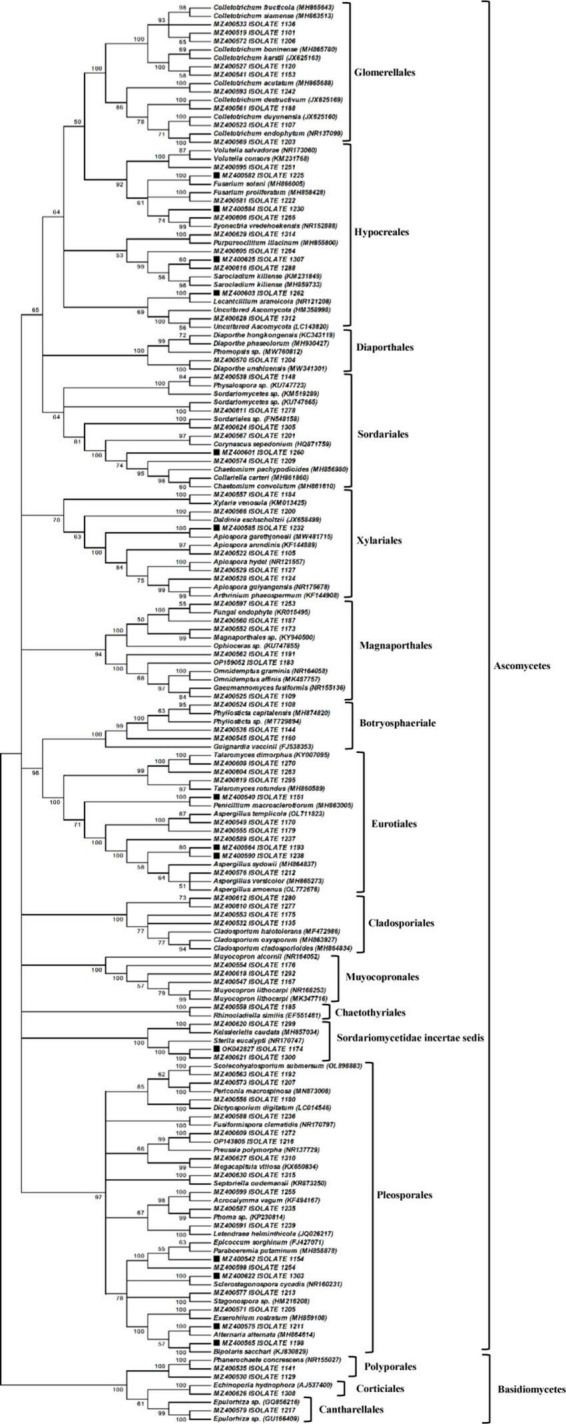
Neighbor-joining (NJ) analysis revealing the phylogenetic relationships of endophytic fungi associated with *Nervilia fordii*. The NJ phylogenetic tree without outside groups includes a total of 169 fungal sequences, in which 85 sequences from our study, 84 reference sequences of close relatives from the NCBI database. Bootstrap support values are indicated for major nodes having values ≥50%. The names of 16 different groups are shown in bold letter such as: Glomerellales, Hypocreales, Diaporthales, Sordariales, Magnaporthales, Xylariales, Botryosphaeriale, Eurotiales, Cladosporiales, Muyocopronales, Chaetothyriales, Sordariomycetidae incertae sedis, Pleosporales, Polyporales, Corticiales, and Cantharellales. The solid square (■) represents the isolates with antimicrobial activity.

A total of 184 culturable endophytic fungi were isolated from the leaves, roots, and corm of *N. fordii*. Among them, 85 different morphotypes (Supplementary Table 1) were recognizable according to their fungal morphological characteristics and used for phylogenetic reconstruction. The phylogenetic analysis was performed using the MEGA program, and the resulting NJ phylogenetic tree is shown in Figure 2; the tree was constructed from an ITS rDNA dataset comprising all morphotype sequences obtained in this study and the GenBank sequences of available close relatives. The resulting phylogenetic tree showed that the endophytic fungi of *N. fordii* belonged to a richly diverse group including Ascomycetes (95.29%) and Basidiomycetes (4.71%). Among the Ascomycetes groups, three classes (Dothideomycetes, Eurotiomycetes, and Sordariomycetes) and 13 orders (Diaporthales, Hypocreales, Magnaporthales, Sordariales, Glomerellales, Xylariales, Chaetothyriales, Eurotiales, Pleosporales, Cladosporiales, Muyocopronales, Botryosphaeriales, and *Sordariomycetidae incertae sedis*) were identified. Other groups involving Cantharellales, Corticiales, and Polyporales were placed within Agaricomycetes of Basidiomycetes.

Further analysis of the tree (Figure 2) showed that Pleosporales was the dominant fungal order of *N. fordii*. Among the Pleosporales, 18 isolates and 17 reference taxa formed a clade with 97% bootstrap support, and further formed six subclades. In the first subclade, three morphologically distinct isolates, i.e., 1192#, 1207#, and 1180#, clustered to *Scolecohyalosporium submersum* (OL898883), *Periconia macrospinosa* (MN873008), and *Dictyosporium digitatum* (LC014546) with 100% bootstrap support, respectively. The second subclade only had one isolate 1236#, which clustered with *Fusiformispora clematidis* (NR170797) with 100% bootstrap support; however, there were relatively low sequence similarities (86.64%) between the morphotype and *F. clematidis*. In another subclade, two isolates (1272# and 1216#) clustered with *Preussia polymorpha* (NR137729) with 99% bootstrap support. In the same subclade, isolate 1310# and the reference taxa *Megacapitula villosa* (KX650834) formed a group with 99% bootstrap support. In the fourth subclade, isolate 1315# and *Septoriella oudemansii* (KR873250) shared a subclade with 100% bootstrapping. In the sixth subclade, two isolates, i.e., 1255# and 1235#, clustered together with *Acrocalymma vagum* (KF494167) and *Phoma* sp. (KP230814), respectively, and formed a terminal clade with 98% bootstrapping. Isolate 1239# and the reference species *Letendraea helminthicola* (JQ026217) formed another terminal clade with 100% bootstrap support. Seven isolates clustered in the last subclade of the order Pleosporales, two isolates of which were closely related to *Epicoccum sorghinum* (FJ427071) and *Paraboeremia putaminum* (MH858878) with 99% sequence similarity, respectively, and formed a terminal clade with 100% bootstrap support. Another five isolates, i.e., 1303#, 1213#, 1205#, 1211#, and 1198#, clustered with *Sclerostagonospora cycadis*, *Stagonospora* sp., *Exserohilum rostratum* (MH859108), *Alternaria alternata* (MH864614), and *Bipolaris sacchari* (KJ830829) with 100% bootstrap values and relatively high nucleotide similarities (98.78–100%).

The clade representing the order Glomerellales had nine morphologically distinct isolates (Figure 2) and grouped with the genus *Colletotrichum* with 100% bootstrapping, four of which were closely related to *C. acutatum* (MH865688), *C. destructivum* (JX625169), *C. duyunensis* (JX625160), and *C. endophytum* (NR_137099) with 100% bootstrap support, respectively. In the same clade of Glomerellales, five isolates clustered with *C. camelliae* (MH864126), *C. fructicola* (MH865643), *C. siamense* (MH863513), *C. boninense* (MH865780), and *C. karstii* (JX625163) with 100% bootstrap support and with 99.02–99.64% nucleotide similarity. In another clade, 11 isolates formed the order Hypocreales, in which one isolate clustered to *Volutella*, two isolates to *Fusarium*, and two to *Ilyonectria* with high bootstrap support. In another subclade of Hypocreales, one isolate grouped with *Purpureocillium lilacinum*, three with *Sarocladium kiliense*, one with *Lecanicillium araneicola*, and one with two unidentified fungal species. Within the clade representing the order Diaporthales, isolate 1204# formed a group with *Physalospora* and three reference isolates of *Diaporthe* with 100% bootstrap support. The clade of the order Sordariales was composed of six isolates, one of which grouped with the genus of *Physalospora*, two of which showed similarities with 100% bootstrap support to two unidentified fungi of Sordariomycetes, and three to *Corynascus sepedonium*, *Collariella carteri*, and *Chaetomium pachypodioides* with 100% bootstrap support. In the clade of Xylariales belonging to Sordariomycetes, the six isolates identified in the current study showed high relatedness to four genera, i.e., *Xylaria*, *Daldinia*, *Apiospora*, and *Arthrinium*. Five isolates belonging to the order Magnaporthales formed a clade with 94% bootstrapping and had affinity with *Omnidemptus*, *Gaeumannomyces*, and two unidentified fungi.

In addition, 11 isolates belonged to the Dothideomycetes class and formed a clade composed of three subclades, i.e., Botryosphaeriale, Cladosporiales, and Muyocopronales. Within Botryosphaeriale, three isolates (1108#, 1144#, and 1160#) were identified as being closely related to four different species of the genus *Phyllosticta* and its teleomorph *Guignardia*. In Cladosporiales, four isolates clustered with three different species of the genus *Cladosporium* with 100% bootstrapping. In another subclade, three endophytic and two *Muyocopron* species formed an assembly of Muyocopronales with 100% bootstrap support.

Another 11 fungal isolates were placed in the Eurotiomycetes class within two orders, i.e., Eurotiales and Chaetothyriales, and formed four fungal assemblies with high bootstrap support. The first fungal assembly included three isolates (1270#, 1263#, and 1295#), that were closely related and formed a subclade with *Talaromyces dimorphus* (KY007095) and *T. rotundus* (MH860589). The 1151# isolate closely clustered with *P. macrosclerotiorum* (MH863005) and formed the second fungal assembly with 100% bootstrap support. Six isolates (1170#, 1179#, 1237#, 1193#, 1238#, and 1212#) were confirmed as being closely related to four different species of the genus *Aspergillus*. The clade representing Chaetothyriales was composed of a singleton isolate (1185#) grouped with *Rhinocladiella similis* (EF551461) with 100% bootstrap support. In addition, three isolates (1299#, 1174#, and 1300#) were well placed in a singleton clade with *Keissleriella caudata* (MH857034) and *Sterila eucalypti* (NR_170747) with 100% bootstrap support, albeit with low nucleotide similarity (85.52–89.39%).

Lastly, four isolates were classified as Basidiomycetes and formed a clade comprising three orders with 100% bootstrap support (Figure 2). In the order Polyporales subclade, two isolates grouped with *Phanerochaete concrescens* (NR_155027) with 100% bootstrap support, but only isolate (1141#) displayed high relatedness with *P. concrescens* with 98.19% nucleotide similarities. In the Corticiales order, isolate 1,308 was well placed in a singleton subclade with *Echinoporia* sp. (MH553213). In the Cantharellales order, isolate 1217# clustered with two unidentified species of the genus *Epulorhiza* and formed a subclade with 99% bootstrap support.

### Diversity analysis of fungal endophytic species from *Nervilia fordii*

The results of the current study revealed that the fungal endophytic species isolated from *N. fordii* exhibited rich and diverse characteristics as showed in Table 1. The total fungal isolates and species diversity of the different tissues of *N. fordii* were comparatively researched using multiple analytical indexes, such as species richness (*S*), Camargo’s index (1/*S*), Simpson’s index (*D*), Simpson’s index of diversity (1−*D*), and the Shannon index of diversity (*H*′). First, the number of total fungal isolates collected from the different tissues of *N. fordii* was 103, 39, and 42 for leaves, roots, and corms, respectively. The species richness (*S*) of endophytic fungi from *N. fordii* was 85 species, of which 48 were collected from leaves, 30 from roots, and 22 from corms. The predominant fungal species collected from the leaves and corms of *N. fordii* was *Apiospora hydei* (P*i* = 0.086 and 1/S = 0.012), followed by an undefined fungus of Magnaporthaceae (P*i* = 0.054) from leaves; *Fusarium* sp. (P*i* = 0.043) from leaves, roots, and corms; *Aspergillus sydowii* (P*i* = 0.038) from corms; and *C. karstii* (P*i* = 0.032) from leaves. Other isolates, i.e., *E. sorghinum*, *Omnidemptus* sp., *Epulorhiza* sp., *A. arundinis*, *Arthrinium* sp., *C. fructicola*, *F. proliferatum*, *Muyocopron lithocarpi*, and *Phoma* sp., were also common in the tissues of *N. fordii*. Our results showed that the highest endophytic population diversities were found in the leaves (1 − D = 0.953; *H*′ = 3.485), followed by the roots (1 − D = 0.953; *H*′ = 3.247), and the corms (1 − D = 0.926; *H*′ = 2.846). Moreover, the diversity index of the fungal community of *N. fordii* was confirmed by diversity values of *H*′ = 4.248 and 1 − D = 0.974.

**TABLE 1:** The diversity of culturable endophytic fungi isolated from the health tissues of *Nervilia fordii*.

Index of diversity	Tissues			Total isolates
		
	Leaves	Roots	Corms	
No. of total fungal isolates	103	39	42	184
Species richness (*S*)	48	30	22	85
Camargo’s index (1/*S*)	0.021	0.033	0.045	0.012
Simpson’s index (*D*)	0.047	0.047	0.074	0.026
Simpson’s index of diversity (1−*D*)	0.953	0.953	0.926	0.974
Shannon index of diversity (*H*′)	3.485	3.247	2.846	4.248

### Antimicrobial activity of endophytic fungi

**FIGURE 3:**
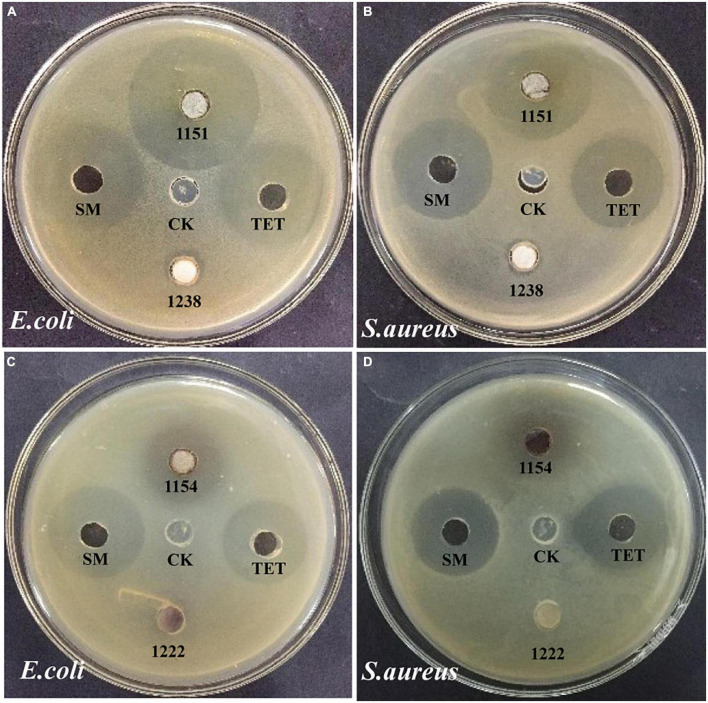
Antimicrobial activity of the fungal endophyte agar plugs of *Penicillium macrosclerotiorum* 1151# and *Epicoccum sorghinum* 1154# against *Escherichia coli* **(A,C)** and *Staphylococcus aureus* **(B,D)** by agar well diffusion method. Dimethyl sulfoxide (DMSO) was used as the blank control (CK); Tetracycline (TET), and Streptomycin (SM) were used as positive control.

The other research aim of this study was to screen for the antimicrobial activity of all endophytic fungi from *N. fordii* using *S. aureus*, *E. coli*, and *C. tropicalis* as targets *via* the agar diffusion method. The results obtained are provided in Table 2. Eight fungal species (9.41%) displayed antibacterial activity against *E. coli*, whereas 11 (12.94%) had activity against *S. aureus* and two (2.35%) against *C. tropicalis*. Interestingly, eight isolates displayed not only antagonistic action toward *S. aureus*, but also antibacterial activity against *E. coli*. In particular, *P. macrosclerotiorum* 1151# showed efficient activity against *E. coli*, providing an inhibition of up to 1.32- and 1.53-fold (Figure 3A) compared with SM and TET, respectively. Moreover, this strain 1151# was also the most efficient in inhibiting *S. aureus*, up to 1.13- and 1.21-fold (Figure 3B) compared with SM and TET, respectively. Other fungal species, i.e., *E. sorghinum* 1154#, *B. sacchari* 1198#, and *A. versicolor* 1238#, were also effective in inhibiting the growth of Gram-negative (Figure 3C) and Gram-positive (Figure 3D) pathogens, although their antibacterial effects were weaker than those of the control. In addition, compared with SM and TET, *Fusarium* sp. 1225# and *S. cycadis* 1303# showed a wider antimicrobial spectrum to inhibit significantly the growth of both pathogenic bacteria and fungi.

**TABLE 2:** Antimicrobial activity of 14 endophytic fungi isolated from *Nervilia fordii* against human pathogenic microorganisms by agar well diffusion method.

Isolate No.	Classification	GenBank No.	Closest match (GenBank No.)−% seq identity	Antimicrobial activity^a^		
				*Escherichia coli*	*Staphylococcus aureus*	*Candida tropicalis*
1,151	*P. macrosclerotiorum*	MZ400540	*P. macrosclerotiorum* (MH863005.1)−99.06%	34.70 ± 3.36	28.48 ± 0.74	–
1,154	*E. sorghinum*	MZ400542	*E. sorghinum* (FJ427071.1)−99.57%	22.12 ± 0.78	15.70 ± 1.09	–
1,174	*Sordariomycetes* sp.	OK042827	*S. eucalypti* (NR_170747.1)−89.39%	–	9.66 ± 0.34	–
1,198	*B. sacchari*	MZ400565	*B. sacchari* (KJ830829.1)−99.27%	12.06 ± 2.08	12.52 ± 0.86	–
1,211	*A. alternata*	MZ400575	*A. alternata* (MH864614.1)−100%	–	8.67 ± 0.19	–
1,225	*F. oxysporum*	MZ400582	*F. oxysporum* (JF807397.1)−99.43%	–	9.95 ± 0.54	12.11 ± 0.63
1,230	*T. blackeriella*	MZ400584	*T. blackeriella* (NR_159037.1)−99%	9.96 ± 0.04	–	–
1,232	*Arthrinium* sp.	MZ400585	*A. subroseum* (NR_157471.1)−97.24%	–	8.74 ± 0.55	–
1,238	*A. versicolor*	MZ400590	*A. versicolor* (MH865273.1)−99.62%	11.08 ± 1.02	10.27 ± 0.82	–
1,193	*A. amoenus*	MZ400564	*A. amoenus* (OL772676.1) −99.81%	8.52 ± 0.46	–	–
1,260	*Collariella* sp.	MZ400601	*C. carteri* (MH861860.1)−96.17%	–	8.73 ± 0.24	–
1,262	*Lecanicillium* sp.	MZ400603	*L. araneicola* (NR_121208.1)−96.43%	9.14 ± 0.61	9.04 ± 0.71	–
1,303	*S. cycadis*	MZ400622	*S. cycadis* (MH863303.1)−99.85%	11.08 ± 0.87	–	9.84 ± 0.55
1,307	*S. kiliense*	MZ400625	*S. kiliense* (MH859733.1)−99.25%	–	10.58 ± 1.70	–
Streptomycin (5 mg ml^–1^, 20 μL)				24.477 ± 2.48	23.34 ± 1.85	–
Tetracycline (5 mg ml^–1^, 20 μL)				26.44 ± 1.74	24.85 ± 3.13	–

^a^Antimicrobial activity illustrated by diameter of inhibition zone, “–” means antibacterial activity not been detected. Streptomycin (SM) and Tetracycline (TET) are used as the positive control.

These results also indicated that fungal species with antimicrobial activities were mainly collected from the roots of *N. fordii*, and were classified into six clades, i.e., Pleosporales, Hypocreales, Eurotiales, Xylariales, Sordariales, and *Sordariomycetidae incertae sedis* (Figure 2 and Supplementary Table 1).

### Determination of minimum inhibitory concentration

**FIGURE 4:**
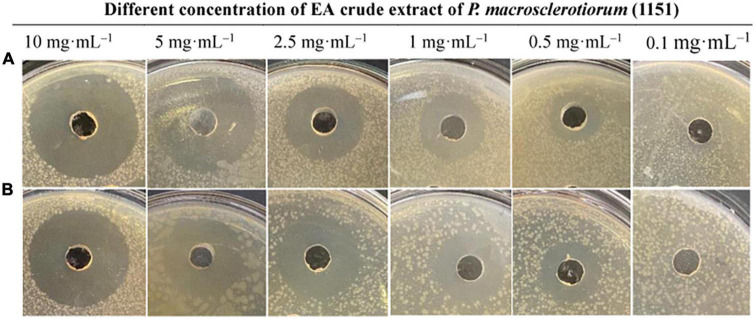
Antimicrobial activity of different concentration of the ethyl acetate (EA) crude extract of *Penicillium macrosclerotiorum* 1151# against *Escherichia coli* **(A)** and *Staphylococcus aureus* **(B)** by agar well diffusion method.

During the antimicrobial assays of fungal agar plug, only *P. macrosclerotiorum* 1151# showed potent activity against Gram-negative and Gram-positive bacteria as having an inhibition zone of 34.70 ± 3.36 and 28.48 ± 0.74 mm (Table 2). This suggested that the *P. macrosclerotiorum* 1151# may be explored as a microbial factory of great potential antibacterial ingredients for industrial applicactions. Therefore, this endophytic fungi has been employed to determine the MIC values of crude extracts by using agar well diffusion method. Interestingly, only the EA crude extracts showed a strong inhibitory activity against tested Gram-negative and Gram-positive bacteria, whereas the PET crude extracts exhibited few growth inhibition against tested organisms as well as *n*-BuOH crude extracts. Moreover, the EA course extracts of *P. macrosclerotiorum* 1151# still had a clear zone of antibacterial activity against *S. aureus* and *E. coli* with MIC at 0.5 mg⋅ml^–1^. The diameters of inhibition zones are showed in Table 3 and illustrated in Figure 4. These results suggested that most antibacterial active fraction of *P. macrosclerotiorum* 1151# were extracted from the EA fraction, but not PET and *n*-BuOH fractions.

**TABLE 3:** Antimicrobial activity as indicated by growth-inhibition zone of different concentration of ethyl acetate (EA) crude extract of *Penicillium macrosclerotiorum* 1151# against gram-negative and gram-positive bacteria.

Bacteria strains	Inhibition zone (mm)					
	
	Different concentration of EA crude extract					
	
	10 mg mL^–1^	5 mg mL^–1^	2.5 mg mL^–1^	1 mg mL^–1^	0.5 mg mL^–1^	0.1 mg mL^–1^
*Escherichia coli*	27.47 ± 0.24	23.58 ± 0.48	21.55 ± 0.14	16.64 ± 0.89	13.22 ± 0.61	–
*Staphylococcus aureus*	25.01 ± 0.27	20.14 ± 0.87	20.1 ± 0.05	14.61 ± 1.29	14.94 ± 0.38	–

“–” means antibacterial activity not been detected.

### Characterization of the purified compound

**FIGURE 5:**
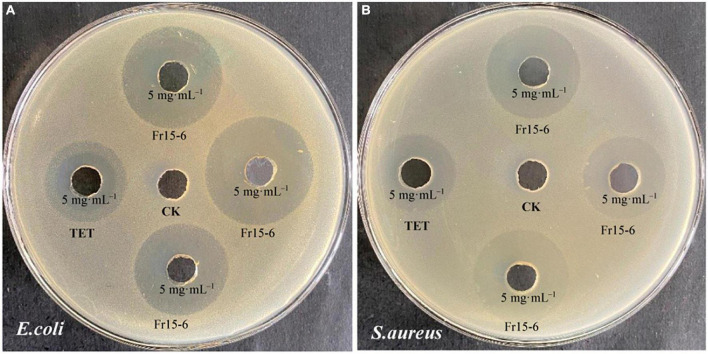
Antimicrobial activity of the monomer component (Fr_15–6_) isolated from the ethyl acetate (EA) crude extract of *Penicillium macrosclerotiorum* 1151# against *Escherichia coli* **(A)** and *Staphylococcus aureus* **(B)** by agar well diffusion method. Dimethyl sulfoxide (DMSO) was used as the blank control (CK), and the tetracycline (TET) as positive control. The concentration of all samples was 5 mg ml^– 1^. The sample of the monomer component (Fr_15–6_) was repeated for three wells in each Petri-dish plate.

**FIGURE 6:**
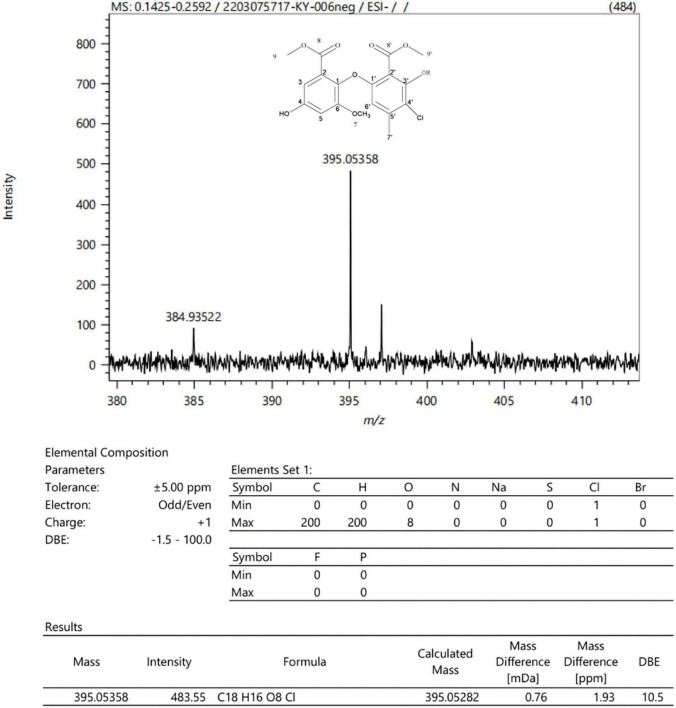
ESI-MS chromatogram and molecular formula of the monomer component isolated from the ethyl acetate (EA) crude extracts of *Penicillium macrosclerotiorum* 1151#.

In order to analyze the most antibacterial active components of *P. macrosclerotiorum* 1151#, 5.64 kg of the fungal rice medium was extracted with 10-fold (w/v) EA, and about 35.81 g of crude extract was obtained. The EA crude extract was chromatographed on a D_101_ macroporous resin column using a gradient of PET:acetone (30:1, 15:1, 3:1, 1:1, and 0:1), to yield 18 fractions (Fr_1_–Fr_18_). However, only the Fr13, Fr14, and Fr15 fractions displayed higher antibacterial activity against *S. aureus* and *E. coli*. Among them, the Fr15 fractions showed a strongest inhibitory activity against tested organisms, and consequently were separated into 12 subfractions (Fr_15–1_–Fr_15–12_) *via* silica gel CC, with elution using PET:EA (10:1–0:1). The results of the antimicrobial assays (Figure 5) suggested that the Fr_15–6_ subfraction was main active compound, which was recorded against *E. coli* and *S. aureus* as having an inhibition zone of 27.53 ± 1.65 and 23.33 ± 2.36 mm, up to 1.71- and 1.13-fold compared with tetracyclin, respectively. Finally, the Fr_15–6_ subfraction was further purified by semi-preparative HPLC (MeOH:H_2_O, 1:1, v/v) and obtain one compound. The chemical structure of this monomer component was confirmed as methyl chloroacetate by analyzing the ESI-MS (Figure 6) and NMR data (Supplementary Table 2 and Supplementary Figures 1–3). All data presented above were consistent with methyl chloroacetate reported in previous studies (Liu et al., 2015; Said et al., 2018).

## Discussion

Antibiotic resistance has become a serious problem threating global public health (Malhadas et al., 2017; Wang et al., 2021), suggesting roles of new antimicrobial drugs in solving this increasingly serious problem in addition to the proper use of antibiotics. Unfortunately, it has become increasingly difficult to find new antibiotics from the conventional soil environment, resulting in nearly three decades without a new antibiotic (Böttcher et al., 2021). Endophytes are being used as an increased important resource for searching new natural antimicrobial compounds, due to their characteristics of wide distribution, species diversity, and the potential to yield novel metabolites (Rajivgandhi et al., 2018b). In this study, we report the rich diverse endophytic fungi have been isolated from the karst endemic plants *N. fordii*. Our *in vitro* antimicrobial assays using the well diffusion methods demonstrated that the fungal agar plugs of some strains can show potent activity against Gram-negative and Gram-positive bacteria. More importantly, for the first time, our study found that the EA crude extracts of *P. macrosclerotiorum* 1151# had stronger antibacterial effects against *E. coli* and *S. aureus*. Notably, a monomer component seperated from the EA fractions of this isolate 1151# was firstly identified as the methyl chloroacetate, which displaying a stronger inhibitory activity against all Gram-negative and Gram-positive bacteria, compared with tetracyclin. These findings provide evidence that various endophytic fungi of *N. fordii* could be exploited as potential sources of novel natural antimicrobial agents.

These endophytic fungi isolated from *N. fordii* were represented by mostly Ascomycetes and relatively small number of Basidiomycetes, in consistent with the results of previous studies on the endophytic fungi of plants (Macia-Vicente et al., 2008; Pecundo et al., 2021). In our study, we described the first time that *Apiospora* sp. was firstly reported as the dominant species of *N. fordii*, followed by *Colletotrichum* sp., *Aspergillus* sp., and *Fusarium* sp. Previously, it was reported that *Colletotrichum* (Huang et al., 2008), *Aspergillus* (Naik et al., 2021), and *Fusarium* (Tejesvi et al., 2011) are the dominant endophytic fungi of plants. In addition, widespread species, such as *Chaetomium*, *Penicillium*, *Phoma*, *Xylaria*, *Phyllosticta*, and *Epicoccum*, have been reported as being endophytic fungi of many plants and were mainly isolated from the leaves and roots (Arnold, 2007; Tejesvi et al., 2011); all were reported in *N. fordii* here for the first time. However, previous study reported a total of 23 strains belonging only a genus *Colletotrichum* isolated from *N. fordii* in the Daxing county of Guangxi Province (Song et al., 2019). Interestingly, most of endophytic fungal genera in the current study were not previously isolated from *N. fordii*, suggesting a unique fungal community with high abundance and rich diversity existed naturally in the roots, leaves, and corms of the karst endemic *N. fordii* plants, compared with the study reported by Song et al. (2019).

In the current study, 15 genera, i.e., *Epulorhiza*, *Echinoporia*, *Thelonectria*, *Septoriella*, *Scolecohyalosporium*, *Sclerostagonospora*, *Sarocladium*, *Purpureocillium*, *Paraboeremia*, *Ilyonectria*, *Exserohilum*, *Daldinia*, *Corallomycetella*, and *Collariella*, were only found in the roots of *N. fordii*, and were absent from its leaves and corms. *Epulorhiza* has been confirmed as an important mycorrhizal fungus isolated from the roots of many photosynthetic orchids (Kristiansen et al., 2004; McCormick et al., 2004; Tan et al., 2014); however, here, it was reported for the first time as the dominant fungal group of the roots of *N. fordii*. Moreover, 11 fungal genera (*Arthrinium*, *Gaeumannomyces*, *Letendraea*, *Muyocopron*, *Omnidemptus*, *Penicillium*, *Physalospora*, *Rhinocladiella*, *Stagonospora*, *Xylaria*, and *Phanerochaete*) and eight unidentified genera were only detected in the leaves of *N. fordii*, whereas six genera were detected exclusively in the corms (Table 1). In contrast, only five fugal communities, i.e., *Apiospora*, *Epicoccum*, *Phoma*, *Phyllosticta*, and *Talaromyces*) were found among the roots and leaves, and only two genera (*Aspergillus* and *Chaetomium*) were detected in all tissues. These results suggest that our reported endophytic fungal genera have obvious tissue specificity features to some extent, which similar to the previous studies (Xing and Guo, 2011; D’Souza and Hiremath, 2013; Cui et al., 2015; Ming et al., 2022).

Increasing evidence is showing that plants with antimicrobial activities may provide the most viable opportunity to screen out novel fungal endophytes with bioactive compounds (Strobel et al., 2004; Lv et al., 2010; Cui et al., 2015; Malhadas et al., 2017). For example, the endophytic *A. alternata* isolated from the leaves of the medicinal plant *Ziziphus spina-christi* displayed positive inhibition activity against several pathogenic bacteria and fungi (Elghaffar et al., 2022). Similarly, endophytic fungi isolated from many plants, i.e., *Dysosma versipellis*, *Saussurea involucrate*, and *Rhododendron tomentosum* (Lv et al., 2010; Tejesvi et al., 2011; Tan et al., 2018), with high antimicrobial efficiency also potentially represent new antibacterial compounds. In our study, 14 endophytic fungi with strong antimicrobial activities were identified in the healthy tissues of *N. fordii* plants and exhibited high antimicrobial capacities, as determined using the agar well diffusion method. These isolates showed an abundant diversity of species, including *Penicillium*, *Epicoccum*, *Bipolaris*, *Alternaria*, *Fusarium*, *Arthrinium*, *Thelonectria*, *Lecanicillium*, *Sclerostagonospora*, Sordariomycetes sp., and *Corallomycetella* (Table 2). For instance, the EA crude extracts of *E. sorghinum* 1154# exerted a strong inhibitory activity against *E. coli* and *S. aureus*. A previous study had reported that *E. sorghinum* L28 had a strong inhibitory effect against the growth of plant pathogenic fungi (Feng et al., 2022). In particular, the 1151# isolate obtained from the leaves of *N. fordii* had a high nucleotide similarity (99.06%) with *P. macrosclerotiorum* (CBS 116871; MH863005), and showed highest antimicrobial activities against *E. coli* and *S. aureus* with a MIC of 0.5 mg ml^–1^. In fact, previous studies had revealed that several fungal species of the genus *Penicillium* can produce various antimicrobial compounds (Nicoletti et al., 2014; Malhadas et al., 2017). The endophytes *P. canescens* and *P. commune* showed antimicrobial activities against to the human pathogens (Gao et al., 2011; Malhadas et al., 2017) and the phytopathogenic fungi (Bertinetti et al., 2009). Other species, such as *P. cinnamopurpureum* (Dinesh et al., 2022), *P. chrysogenum* (Orfali et al., 2022), and *P. citrinum* (Kumari et al., 2021), had also been reported as being prolific producers of antimicrobial compounds. However, no studies have reported the antibacterial activity of *P. macrosclerotiorum*, which was identified as a new species of the genus *Penicillium* in 2017 (Wang et al., 2007; Xu et al., 2021). Our study reported for the first time the strong antimicrobial potential of EA crude extracts of *P. macrosclerotiorum* against Gram-negative and Gram-positive pathogenic bacteria compared with two commercial antibiotics. Furthermore, our study also confirmed that an antimicrobial component, i.e., methyl chloroacetate, which had been previously isolated from two coastal saline soil fungi [*A. iizukae* (Liu et al., 2015) and *Aspergillus* sp. (Said et al., 2018)], was detected in *P. macrosclerotiorum* for the first time. However, due to insufficient sample, the antimicrobial compounds of other fractions in the current study needs to be clarified in the future, as well as the MIC values of methyl chloroacetate, etc.

## Conclusion

We firstly concludes that an unique and diverse cultivable endophytic fungal assemblage inhabiting the roots, corms, and leaves of karst endemic *N. fordii* plants. We show that the fungal communities displayed obvious tissue specificity features and may be closely related to the different function of organizations as well as the external conditions. The endophytic fungus *P. macrosclerotiorum* 1151# isolated from the leaves of *N. fordii* exhibit the high ability to inhibit the growth of the tested Gram-positive and Gram-negative bacteria. In fact, the current study confirm, for the first time, one of the antibacterial active substances within the EA crude extracts of *P. macrosclerotiorum* 1151# was methyl chloroacetate through Semi-preparative HPLC and NMR analyses. Therefore, our studies provide a basis for the exploration of new natural antimicrobial agents from medicinal plants with antimicrobial function in the future, which may contribute to the solution of worldwide antimicrobial resistance in the long run.

## Data availability statement

The datasets presented in this study can be found in online repositories. The names of the repository/repositories and accession number(s) can be found in the article/Supplementary material.

## Author contributions

Y-QZ, X-MT, and RS-H designed the research. X-FY, X-YX, and Y-QZ performed the experiments. S-CY, YT, and JW analyzed the data. X-MT, L-YY, and PF collected the samples. X-MT, S-CY, and Y-QZ co-wrote the manuscript. All authors contributed to the manuscript and approved the submitted version.
