# Supplementary material for: Cultivable endophytic fungal community associated with the karst endemic plant Nervilia fordii and their antimicrobial activity
Source: Front Microbiol. 2022 Nov 24;13:1063897. doi: 10.3389/fmicb.2022.1063897 (PMC9730403; doi:10.3389/fmicb.2022.1063897)
Supplement: Supplementary file 1 [file Data_Sheet_1.docx]

Supplementary Material

**Cultivable Endophytic Fungal Community**

**Associated with The Karst Endemic Plant *Nervilia fordii***

**and Their Antimicrobial Activity**

**Ya-Qin Zhou^1,2†^, Shao-Chang Yao^1†^, Jie Wang^1,3^, Xiao-Ming Tan^1*^, Xin-Yi Xie^1^, Rong-Shao Huang^1^, Xin-Feng Yang^1^, Yong Tan^1^, Li-Ying Yu^2^ and Peng Fu^1,3^**

^1^ College of Pharmacy, Guangxi University of Chinese Medicine, Nanning, China

^2^ Guangxi Key Laboratory of Medicinal Resources Conservation and Genetic Improvement, Guangxi Botanical Garden of Medicinal Plant, Nanning, China

^3^ Guangxi Zhuang Yao Key Laboratory of Medicine, Guangxi University of Chinese Medicine, Nanning, China

***Correspondence:**

Xiaoming Tan (tanxm@gxtcmu.edu.cn)

**^†^**These authors have contributed equally to this work.

# Supplementary Tables

**1.1 Supplementary Table S1 Classification, distribution and accession number of endophytic fungi from *Nervilia fordii***

| **Taxon (Accession number)** | **Tissues** | | | **Total isolates** | **Represen-tative isolates** | **Genbank closest match (accession no.)** | **Identity*^a^* (%)** |
| --- | --- | --- | --- | --- | --- | --- | --- |
|  | **Leaves** | **Roots** | **Corms** |  |  |  |  |
| **Ascomycetes** |  |  |  |  |  |  |  |
| *Acrocalymma vagum* (MZ400599) |  |  | 3 | 3 | 1255 | *Acrocalymma vagum* (KF494167) | 99.20% |
| *Alternaria alternata* (MZ400575) |  |  | 2 | 2 | 1211 | *Alternaria alternata* (MH864614) | 100% |
| *Apiospora arundinis* (MZ400522) | 3 | 1 |  | 4 | 1105 | *Apiospora arundinis* (KF144889) | 99.78% |
| *A. hydei* (MZ400529) | 14 | 2 |  | 16 | 1127 | *Apiospora hydei* (NR_121557) | 98.64% |
| *Arthrinium* sp. (MZ400528) | 4 |  |  | 4 | 1124 | *Arthrinium phaeospermum* (MH857420) | 96.97% |
| *Arthrinium subroseum* (MZ400585) | 2 |  |  | 2 | 1232 | *Arthrinium subroseum* (NR_157471) | 97.24% |
| Aspergillaceae sp. (MZ400576) | 1 |  |  | 1 | 1212 | *Aspergillus amoenus* (OL772676) | 93.05% |
| *Aspergillus* sp. (MZ400555) | 1 | 1 |  | 2 | 1179 | *Aspergillus templicola* (OL711823) | 95.74% |
| *A. amoenus* (MZ400564) | 1 | 1 | 1 | 3 | 1193 | *Aspergillus amoenus* (OL772676) | 99.81% |
| *A. sydowii* (MZ400589) |  |  | 7 | 7 | 1237 | *Aspergillus sydowii* (MH864837) | 100% |
| *A. templicola* (MZ400549) |  | 1 |  | 1 | 1170 | *Aspergillus templicola* (NR_135456) | 99.64% |
| *A. versicolor* (MZ400590) | 1 | 1 |  | 2 | 1238 | *Aspergillus versicolor* (MH865273) | 99.62% |
| *Bipolaris sacchari* (MZ400565) |  | 2 |  | 2 | 1198 | *Bipolaris sacchari* (KJ830829) | 99.27% |
| *Chaetomium globosum* (MZ400567) |  |  | 1 | 1 | 1201 | *Chaetomium globosum* (MH856790) - | 100% |
| *C. pachypodioides* (MZ400574) |  | 2 |  | 2 | 1209 | *Chaetomium pachypodioides* (MH856980) | 99.61% |
| *C. cladosporioides* (MZ400532) | 1 |  |  | 1 | 1135 | *Cladosporium cladosporioides* (MH864834) | 99.20% |
| *C. halotolerans* (MZ400612) |  |  | 1 | 1 | 1280 | *Cladosporium halotolerans* (MF472986) | 99.04% |
| *Cladosporium oxysporum* (MZ400553) | 1 |  |  | 1 | 1175 | *Cladosporium oxysporum* (MH863927) | 99.41% |
| *Cladosporium* sp. (MZ400610) |  |  | 1 | 1 | 1277 | *Cladosporium sphaerospermum* (DQ780343) | 96.34% |
| *Collariella* sp. (MZ400601) |  | 1 |  | 1 | 1260 | *Collariella carteri* (MH861860) | 96.17% |
| *Colletotrichum acutatum* (MZ400593) |  | 1 |  | 1 | 1242 | *Colletotrichum acutatum* (MH865688) | 99.08% |
| *C. boninense* (MZ400527) | 2 |  |  | 2 | 1120 | *Colletotrichum boninense* (MH865780) | 99.59% |
| *C. camelliae* (MZ400533) | 1 |  |  | 1 | 1136 | *Colletotrichum camelliae* (MH864126) | 99.63% |
| *C. destructivum* (MZ400561) | 2 |  |  | 2 | 1188 | *Colletotrichum destructivum* (JX625169) | 99.27% |
| *C. duyunense* (MZ400523) | 1 |  |  | 1 | 1107 | *Colletotrichum duyunensis* (JX625160) | 99.81% |
| *C. endophytum* (MZ400569) |  | 1 |  | 1 | 1203 | *Colletotrichum endophytum* (NR_137099) | 99.81% |
| *C. fructicola* (MZ400519) | 4 |  |  | 4 | 1101 | *Colletotrichum fructicola* (MH865643) | 99.63% |
| *C. karstii* (MZ400541) | 6 |  |  | 6 | 1153 | *Colletotrichum karstii* (JX625163) | 99.64% |
| *C. siamense* (MZ400572) | 1 |  |  | 1 | 1206 | *Colletotrichum siamense* (MH863513) | 99.02% |
| *Corallomycetella repens* (MZ400625) |  | 1 |  | 1 | 1307 | *Corallomycetella repens* (MT267825) | 99.63% |
| *Daldinia eschscholtzii* (MZ400566) |  | 1 |  | 1 | 1200 | *Daldinia eschscholtzii* (JX658499) | 99.0 % |
| *Diaporthe* sp. (MZ400570) |  | 1 | 2 | 3 | 1204 | *Diaporthe longicolla* (HM347700) | 98.0% |
| *Dictyosporium digitatum* (MZ400556) |  |  | 2 | 2 | 1180 | *Dictyosporium digitatum* (LC014546) | 99.61% |
| Pleosporales sp. (MZ400627) |  |  | 1 | 1 | 1310 | *Megacapitula villosa* (KX650834) | 87.60% |
| *Epicoccum sorghinum* (MZ400542) | 1 | 4 |  | 5 | 1154 | *Epicoccum sorghinum* (FJ427071) | 99.57% |
| Eurotiales sp. (MZ400619) |  |  | 1 | 1 | 1295 | *Talaromyces rotundus* (MH860589) | 90.24% |
| *Exserohilum rostratum* (MZ400571) |  | 1 |  | 1 | 1205 | *Exserohilum rostratum* (MH859108) | 99% |
| *Fusarium proliferatum* (MZ400581) | 3 |  | 1 | 4 | 1222 | *Fusarium proliferatum* (MH858428) | 99.21% |
| *F. oxysporum* (MZ400582) | 1 | 2 | 5 | 8 | 1225 | *Fusarium oxysporum* (R997536) | 99.62% |
| *Gaeumannomyces* sp. (MZ400525) | 1 |  |  | 1 | 1109 | *Gaeumannomyces fusiformis* (NR_155136) | 97.87% |
| *Ilyonectria vredehoekensis* (MZ400606) |  | 2 |  | 2 | 1265 | *Ilyonectria vredehoekensis* (NR_152888) | 99% |
| *Lecanicillium* sp. (MZ400603) |  |  | 1 | 1 | 1262 | *Lecanicillium araneicola* (NR_121208) | 96.43% |
| *Letendraea helminthicola* (MZ400591) | 1 |  |  | 1 | 1239 | *Letendraea helminthicola* (JQ026217) | 98.4% |
| Magnaporthaceae sp. (OP159052) | 10 |  |  | 10 | 1183 | *Omnidemptus graminis* (MK487758) | 90.47% |
| *Muyocopron lithocarpi* (MZ400618) | 4 |  |  | 4 | 1292 | *Muyocopron lithocarpi* (MK347716) | 99.25% |
| *Muyocopron* sp. (MZ400554) | 1 |  |  | 1 | 1176 | *Muyocopron lithocarpi* (NR_168253) | 95.78% |
| Muyocopronales sp. (MZ400547) | 1 |  |  | 1 | 1167 | *Muyocopron alcornii* (NR_164052) | 91.69% |
| *Omnidemptus* sp. (MZ400562) | 5 |  |  | 5 | 1191 | *Omnidemptus affinis* (MK487757) | 95% |
| *Paraboeremia putaminum* (MZ400598) |  | 2 |  | 2 | 1254 | *Paraboeremia putaminum* (MH858878) | 99.6% |
| *Penicillium macrosclerotiorum* (MZ400540) | 1 |  |  | 1 | 1151 | *Penicillium macrosclerotiorum* (MH863005) | 99.06% |
| *Periconia macrospinosa* (MZ400573) |  |  | 1 | 1 | 1207 | *Periconia macrospinosa* (MN873008) | 99.43% |
| *Phoma* sp. (MZ400587) | 1 |  | 3 | 4 | 1235 | *Phoma* sp. (KP230814) | 97.28% |
| *Phyllosticta capitalensis* (MZ400524) | 1 | 1 |  | 2 | 1108 | *Phyllosticta capitalensis* (MT071244) | 99.67% |
| *P. elongata* (MZ400536) | 2 |  |  | 2 | 1144 | *Phyllosticta elongate*（EU167584） | 99.67% |
| *Phyllosticta* sp. (MZ400545) | 3 |  |  | 3 | 1160 | *Phyllosticta* sp. （KP900240） | 98.16% |
| *Physalospora* sp. (MZ400538) | 2 |  |  | 2 | 1148 | *Physalospora* sp. （KU747723） | 98.86% |
| Pleosporales sp. (MZ400588) |  |  | 2 | 2 | 1236 | Pleosporales sp.（MZ400586） | 99.52% |
| *Purpureocillium lilacinum* (MZ400629) |  | 1 |  | 1 | 1314 | *Purpureocillium lilacinum*（MH855800） | 99.64% |
| *Rhinocladiella similis* (MZ400558) | 1 |  |  | 1 | 1185 | *Rhinocladiella similis*（EF551461） | 99.47% |
| *Sarocladium kiliense* (MZ400616) |  | 1 |  | 1 | 1288 | *Sarocladium kiliense*（KM231849） | 99.63% |
| *Sarocladium* sp. (MZ400605) |  | 1 |  | 1 | 1264 | *Sarocladium kiliense*（MH859733） | 99.25% |
| *Sclerostagonospora cycadis* (MZ400622) |  | 1 |  | 1 | 1303 | *Sclerostagonospora cycadis*（NR_160231） | 99.85% |
| *Scolecohyalosporium* sp. (MZ400563) |  | 1 |  | 1 | 1192 | *Scolecohyalosporium submersum*（OL898883） | 96.43% |
| *Septoriella oudemansii* (MZ400630) |  | 1 |  | 1 | 1315 | *Septoriella oudemansii*（KR873250） | 99% |
| Sordariales sp. (MZ400624) | 2 |  |  | 2 | 1305 | Sordariales sp.（FN548158） | 96.89% |
| Sordariomycetes sp. (MZ400621) | 1 |  |  | 1 | 1300 | *Sterila eucalypti*（NR_170747） | 89.39% |
| Sordariomycetes sp. (OK042827) | 1 |  |  | 1 | 1174 | *Sterila eucalypti*（NR_170747） | 89.3% |
| Hypocreales sp. (MZ400628) |  |  | 1 | 1 | 1312 | Papulosa sp.（MH141240） | 86% |
| Sordariomycetes sp. (MZ400620) |  |  | 4 | 4 | 1299 | *Keissleriella caudata*（MH857034） | 85.52% |
| Sordariales sp. (MZ400611) | 1 |  | 1 | 2 | 1278 | Sordariales sp. （MH268093） | 91.05% |
| Magnaporthales sp. (MZ400560) | 1 |  |  | 1 | 1187 | *Ophioceras* sp. （KU747855） | 93.3% |
| Magnaporthales sp. (MZ400552) | 4 |  |  | 4 | 1173 | *Corynascus sepedonium*（HQ871759） | 92.06% |
| Magnaporthales sp. (MZ400597) | 1 |  |  | 1 | 1253 | *Ophioceras* sp.（KU747946） | 93.04% |
| Pleosporales sp. (OP143805) | 1 |  |  | 1 | 1216 | *Preussia polymorpha*（NR_137729） | 93.23% |
| Sporormiaceae sp. (MZ400609) | 1 |  |  | 1 | 1272 | *Preussia polymorpha* （NR_137729） | 93.01% |
| *Stagonospora* sp. (MZ400577) | 2 |  |  | 2 | 1213 | *Stagonospora* sp. （OM337558） | 98.78% |
| *Talaromyces dimorphus* (MZ400608) | 1 |  |  | 1 | 1270 | *Talaromyces dimorphus*（KY007095） | 99.43% |
| *Talaromyces* sp. (MZ400604) | 1 | 1 |  | 2 | 1263 | *Talaromyces fusiformis* （NR_169911） | 98.51% |
| *Thelonectria blackeriella* (MZ400584) |  | 3 |  | 3 | 1230 | *Thelonectria blackeriella*（NR_159037） | 99% |
| *Volutella* sp. (MZ400595) |  |  | 1 | 1 | 1251 | *Volutella consors*（KM231768） | 95.01% |
| *Xylaria* sp. (MZ400557) | 1 |  |  | 1 | 1184 | *Xylaria venosula*（KM013425） | 96.55% |
| **Basidiomycetes** |  |  |  |  |  |  |  |
| Polyporales sp. (MZ400530) |  |  | 1 | 1 | 1129 | *Rhizoctonia* sp. (MZ506756) | 78.22% |
| *Echinoporia* sp. (MZ400626) |  | 1 |  | 1 | 1308 | *Echinoporia* sp. (MH553213) | 97.70% |
| *Epulorhiza* sp. (MZ400579) |  | 5 |  | 5 | 1217 | *Epulorhiza* sp.（GU166409） | 98.31% |
| *Phanerochaete* sp. (MZ400535) | 1 |  |  | 1 | 1141 | *Phanerochaete concrescens*（NR_155027） | 98.19% |

^a^ Identification based on ITS rDNA sequence analysis.

**1.2 Supplementary Table S2 NMR data for the monomer component isolated from EA crude extracts of *P. macrosclerotiorum* （1151）(DMSO-d6, 400 MHz, TMS).**

| **Position** | ***δ_H_*** | ***δ_C_*** | **Position** | ***δ_H_*** | ***δ_C_*** |
| --- | --- | --- | --- | --- | --- |
| 1 |  | 135.89 | 1′ |  | 153.74 |
| 2 |  | 124.85 | 2′ |  | 111.26 |
| 3 | 6.74 (d,2.8) | 107.77 | 3′ |  | 151.08 |
| 4 |  | 154.90 | 4′ |  | 114.03 |
| 5 | 6.54 (d,2.9) | 105.10 | 5′ |  | 138.44 |
| 6 |  | 153.42 | 6′ | 6.51 (s) | 110.93 |
| 7 |  | 56.53 | 7′ | 2.28 (s) | 20.07 |
| 8 |  | 165.07 | 8′ |  | 164.98 |
| 9 | 3.23 (s) | 52.08 | 9′ |  | 52.29 |
| 10 |  |  |  |  |  |
| OH-4 | 9.79 (s) |  |  |  |  |
| OH-2′ |  |  |  |  |  |
| OH-3′ | 9.97 (s) |  |  |  |  |
| OH-6′ |  |  |  |  |  |
