# Supplementary material for: Cultivable endophytic fungal community associated with the karst endemic plant Nervilia fordii and their antimicrobial activity
Source: Front Microbiol. 2022 Nov 24;13:1063897. doi: 10.3389/fmicb.2022.1063897 (PMC9730403; doi:10.3389/fmicb.2022.1063897)
Supplement: Supplementary file 2 [file Data_Sheet_2.pdf]

frontiers

Supplementary Material

Cultivable Endophytic Fungal Community Associated with The Karst Endemic Plant *Nervilia fordii* and Their Antimicrobial Activity

Ya-Qin Zhou[1,2†], Shao-Chang Yao[1†], Jie Wang[1,3], Xin-Yi Xie[1], Xiao-Ming Tan[1*], Rong-Shao Huang[1], Xin-Feng Yang[1], Yong Tan[1], Li-Ying Yu[2] and Peng Fu[1,3]

[1] College of Pharmacy, Guangxi University of Chinese Medicine, Nanning, China

[2] Guangxi Key Laboratory of Medicinal Resources Conservation and Genetic Improvement, Guangxi Botanical Garden of Medicinal Plant, Nanning, China

[3] Guangxi Zhuang Yao Key Laboratory of Medicine, Guangxi University of Chinese Medicine, Nanning, China

***Correspondence:**
Xiaoming Tan (tanxm@gxtcmu.edu.cn)

[†]These authors have contributed equally to this work.

1 Supplementary Figures (A total of 3)

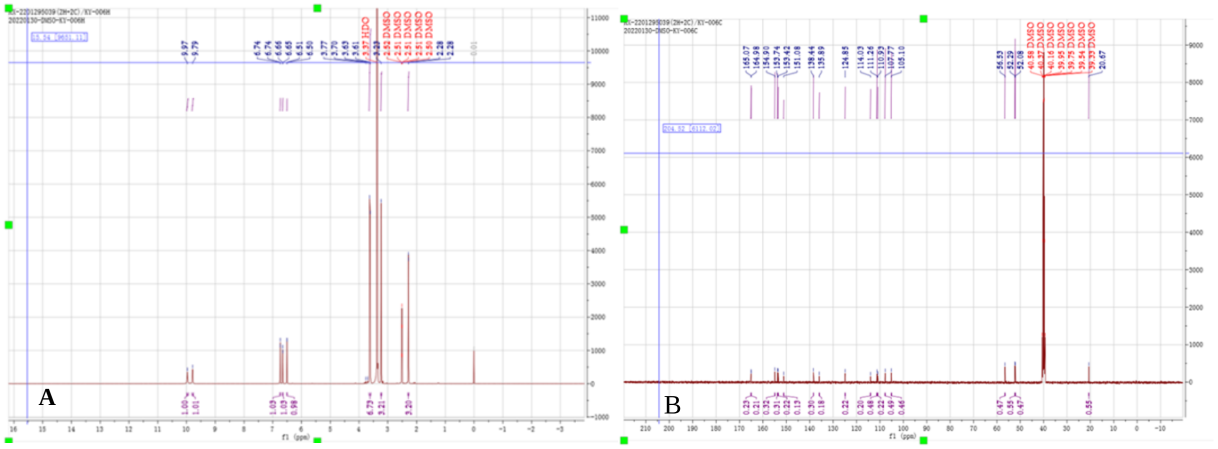

SUPPLEMENTARY FIGURE S1 ^{1}H (A) and ^{13}C (B) NMR chromatogram of the monomer component collected from the EA crude extracts of *P. macrosclerotiorum*

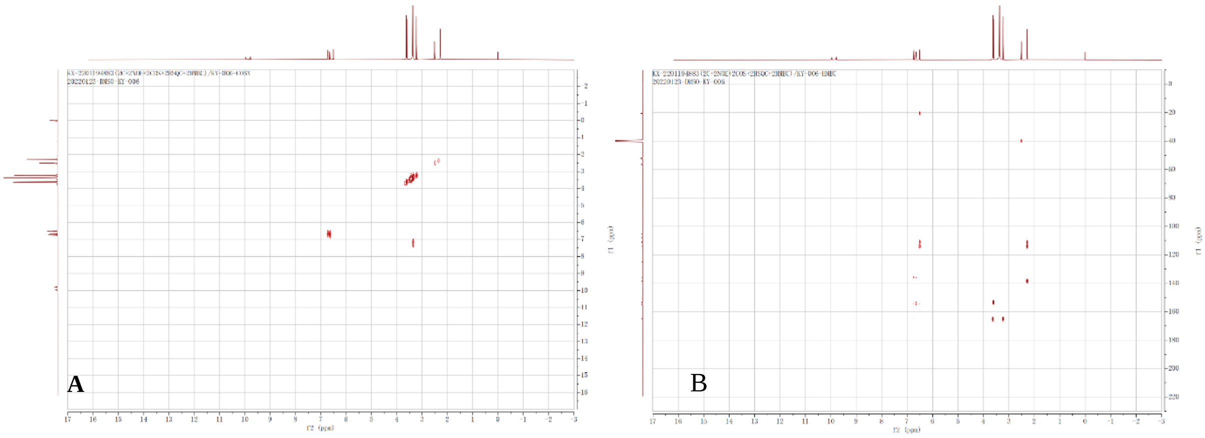

SUPPLEMENTARY FIGURE S2 ^{1}H-^{1}H COSY (A) and HMBC (B) chromatogram of the monomer component collected from the EA crude extracts of *P. macrosclerotiorum*

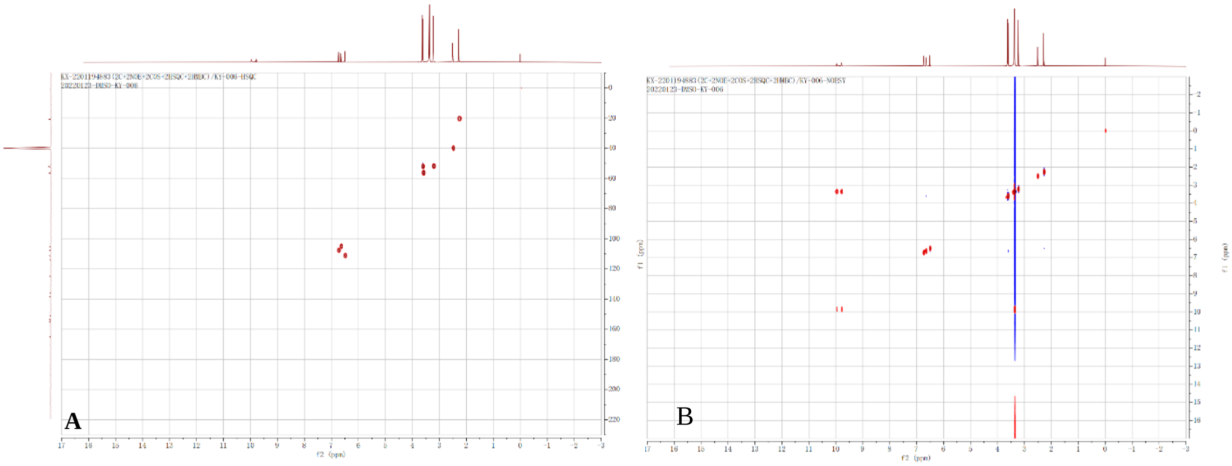

SUPPLEMENTARY FIGURE S3 HSQC (A) and NOESY (B) chromatogram of the monomer component collected from the EA crude extracts of *P. macrosclerotiorum*
